# Immune Factors in Deep Vein Thrombosis Initiation

**DOI:** 10.1016/j.it.2018.04.010

**Published:** 2018-08

**Authors:** Ivan Budnik, Alexander Brill

**Affiliations:** 1 Department of Pathophysiology, Sechenov First Moscow State Medical University, Moscow, Russia; 2 Institute of Cardiovascular Sciences, College of Medical and Dental Sciences, University of Birmingham, Birmingham, UK

## Abstract

Deep vein thrombosis (DVT) is a major origin of morbidity and mortality. While DVT has long been considered as blood coagulation disorder, several recent lines of evidence demonstrate that immune cells and inflammatory processes are involved in DVT initiation. Here, we discuss these mechanisms, in particular, the role of immune cells in endothelial activation, and the immune cascades leading to expression of adhesion receptors on endothelial cells. We analyze the specific recruitment and functional roles of different immune cells, such as mast cells and leukocytes, in DVT. Importantly, we also speculate how immune modulation could be used for DVT prevention with a lower risk of bleeding complications than conventional therapeutic approaches.

## Highlights

Local endothelial activation, and the release of Weibel–Palade bodies present in endothelial cells, are instrumental for deep vein thrombosis (DVT) initiation. In addition to the vascular system, the immune system is emerging as a pivotal player in the pathophysiology of DVT, potentially opening new prophylactic approaches.

Mast cell activation and degranulation are crucial for the local inflammatory response resulting in DVT. Mast cells may potentially link hypoxia and endothelial activation.

Neutrophil extracellular traps play an important role in DVT by providing a scaffold for adhering platelets and red blood cells and promoting thrombin generation and fibrin deposition.

Platelets are an important source of damage-associated molecular patterns facilitating further leukocyte recruitment and promoting venous thrombosis.

## DVT: A Global Health Problem

DVT and its major complication, pulmonary embolism (PE), designated together as venous thromboembolism (VTE), are one of the leading causes of disability and death worldwide. VTE is the third most common cardiovascular pathology by its prevalence after myocardial infarction and stroke [1], with about 900 000 cases and 300 000 deaths in the US annually. Surprisingly, the prevalence and mortality of VTE has not substantially decreased over 30 years despite progress in diagnostic and prophylactic modalities [2]. DVT develops in deep veins, usually, but not exclusively, in legs, causing pain, redness, swelling, and impaired gait. If the thrombus is unstable, it can become detached and travel to the lungs, where it occludes pulmonary circulation causing PE. In contrast to arterial thrombosis, whose mechanisms have been intensively investigated, DVT remains largely *terra incognita*, which inspired the American Surgeon General to issue a Call to Action to stimulate research of venous thrombosis [3].

## Blood Coagulation Cascade and Thrombus Development

**Figure 1:**
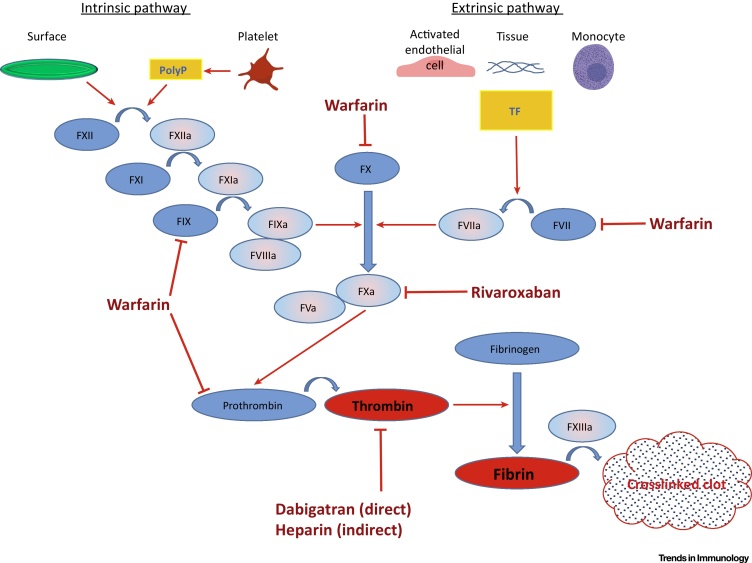
Blood Coagulation Cascade and Major Targets of Current Antithrombotic Therapy. There are two pathways of blood coagulation initiation: intrinsic and extrinsic. The intrinsic pathway starts from activation of FXII by contact with a negatively charged surface, whereas the external one commences with FVII activation by interaction with TF on the surface of monocytes, microparticles, activated endothelial cells, or cells in the injured vessel wall. Both pathways converge on activation of FX (prothrombinase), which converts inactive thrombin precursor prothrombin into active enzyme. Thrombin, a central molecule in the cascade, cleaves fibrinogen turning it into fibrin that consititutes basis for a thrombus. Fibrin crosslinking by active FXIII with subsequent clot retraction make the clot more solid, firm and resistant to fibrin-degrading enzymes. Major targets of the contemporary antithrombotic therapy are depicted. Abbreviations: FVa, activated factor V; FVII, factor VII; FVIIa, activated FVII; FVIIIa, activated factor VIII; FIX, factor IX; FIXa, activated FIX; FX, factor X; FXa, activated FX; FXI, factor XI; FXIa, activated FXI; FXII, factor XII; FXIIa, activated FXII; FXIIIa, activated factor XIII; PolyP, polyphosphate; TF, tissue factor.

Blood clotting is based on a protein polymer called fibrin, produced by cleavage of its precursor, fibrinogen, by the protease thrombin (Figure 1) [4]. Thrombin is formed by activated Factor X (FXa)-mediated processing of prothrombin. Activation of FX can occur via two mechanisms designated as extrinsic and intrinsic pathways. The former one is initiated by a protein designated as tissue factor (TF), which may be exposed by the tissues or blood cells, predominantly monocytes. The intrinsic pathway starts from contact of FXII with a negatively charged surface. Both pathways trigger a cascade of enzymatic transformations converging on FX. Upon formation, fibrin is stabilized by a transglutaminase, FXIII.

Because of the known roles of these factors in clot formation, the current paradigm of DVT prophylaxis focuses predominantly on the coagulation system, by targeting thrombin (e.g., dabigatran), active FXa (e.g., rivaroxaban) or vitamin K-dependent clotting factors (e.g., warfarin; Figure 1). However, due to the substantial overlap in the mechanisms of normal hemostasis and pathological thrombosis, the therapeutic window of anticoagulants may be narrow because of increased chances for bleeding complications. Using a mouse model (Table 1), the Wolberg group has demonstrated an antithrombotic potential of targeting coagulation FXIII, thus preventing retention of red blood cells in the thrombus 5, 6. Given that partial inhibition of FXIII does not impair hemostasis, this may be a promising anti-DVT approach, although its usefulness in humans needs validation (Table 2). More broadly, there is a general demand for a fundamentally new approach that would allow for efficient DVT prevention without risk of bleeding. In this review, we discuss recent advances in understanding the mechanisms of DVT, demonstrating a pivotal role of the immune system in its pathogenesis, and show that recent experimental data call for a paradigm shift, namely to reconsider DVT as an immunity- and inflammation-related process rather than merely coagulation-dependent thrombosis.

**Table 1:** Mouse Models of Venous Thrombosis

Model	Major factor inducing thrombosis	Comments and Disadvantages (D/A)	Refs
IVC stenosis + short external compression	Flow stagnation + endothelial damage	D/A: produces endothelial injury untypical for DVT	8, 105
IVC stenosis	Flow stagnation	Strong inflammatory component in thrombosis initiation D/A: substantial variation in thrombus size	8, 20, 21, 28
IVS stasis	Complete flow cessation	Important role of blood coagulation. D/A: no flow	8, 19, 74
Electrolytic model	Endothelial activation	D/A: breaches the integrity of the IVC wall	8, 106
Ferric chloride	Endothelial denudation	D/A: produces endothelial injury untypical for DVT	8, 107
Rose Bengal/irradiation	Endothelial injury	D/A: produces endothelial injury untypical for DVT	[107]

**Table 2:** Cells and Molecules Involved in Experimental DVT

Targets	Model/effect of genetic deficiency or inhibition or infusion	Refs
Targets supporting DVT		
VWF	Stenosis model/reduced thrombosis prevalence Stasis model/reduced thrombosis prevalence	[21]
P-selectin	Stenosis model/reduced thrombosis prevalence	[28]
E-selectin	Stasis model/smaller thrombi	[108]
CLEC-2	Stenosis model/full conditional knockout, full protection, no thrombi; platelet-specific knockout, lower thrombosis prevalence	[86]
Podoplanin	Stenosis model/smaller thrombi	[86]
Tissue factor	Stasis model/reduced thrombus size in tumor-bearing mice	[109]
PCSK9	Stenosis model/reduced thrombosis prevalence and thrombus size, reduced neutrophil recruitment	[57]
MRP-14	Stasis model/lower thrombus weight Stenosis model/lower thrombosis prevalence	[94]
Complement C3 and C5	Stenosis model/C3: smaller thrombi, lower thrombosis prevalence; C5: smaller thrombi	[36]
apoA-I	Stenosis model/genetic ablation: higher thrombosis prevalence; infusion: lower thrombosis prevalence	[95]
eNOS	Stenosis model/higher thrombosis prevalence	[95]
TLR-4	Stasis model/in endotoxemia: smaller thrombi	[41]
ICAM-1	Stasis model/in endotoxemia: smaller thrombi; in Klebsiella-induced pneumonia: smaller thrombi	41, 110
NETs	Stenosis model/reduced thrombosis prevalence; in APS: reduced thrombosis prevalence	59, 72, 111
HMGB1	Stenosis model/reduced thrombosis prevalence	[65]
mTORC1	Stasis model/smaller thrombi	[87]
Galectin-3	Stasis model/smaller thrombi	[112]
Thromboxane A2	Stasis model/smaller thrombi after aspirin administration	[82]
MCs	Stenosis model/genetic ablation: full protection, pharmacological degranulation inhibition: reduced thrombosis prevalence	[46]
NLRP3	Stasis model/smaller thrombi	[25]
Interleukin-17A	Stenosis model/infusion induces larger thrombi	[113]
Growth arrest-specific 6 (Gas6)	Stenosis model/smaller thrombi	[114]
Coagulation Factor XIII	Stasis model/smaller thrombi	5, 6
Targets inhibiting DVT		
APP	Stasis model/larger thrombi and higher embolization level	[98]
PTGS-2	Stasis model/larger thrombi, higher thrombus firmness and elasticity	[115]
TLR-9	Stasis model/larger thrombi	[73]
SR-BI	Stenosis model/increased thrombosis prevalence	[95]
17α-estradiol	Stasis model/infusion induces smaller thrombi	[116]
Glutathione peroxidase-1	Stasis model/larger thrombi	[52]

Of note, once a thrombus is formed, a process of its resolution begins. In mouse models, thrombus size reaches its maximum within the first 1–2 days, after which it gradually decreases during 2–3 weeks 7, 8, 9. The process of venous thrombus resolution depends on leukocytes, cytokines, metalloproteinases, as well as effector–memory T cells, sharing certain similarities with wound healing 10, 11. When DVT has occurred, even its successful treatment (in many cases surgical, catheter-guided thrombus dissolution) does not preclude all the spectrum of DVT complications, such as recurrence, pulmonary hypertension, post-thrombotic syndrome, and others. Also, in case of a tardy diagnosis, life-threatening PE can develop. Consequently, the primary effort of translational research should focus on DVT prevention and in this review, we have therefore concentrated specifically on the mechanisms of venous thrombosis initiation. For readers interested in thrombus resolution we recommend the following reviews on the topic 12, 13.

**Figure 2:**
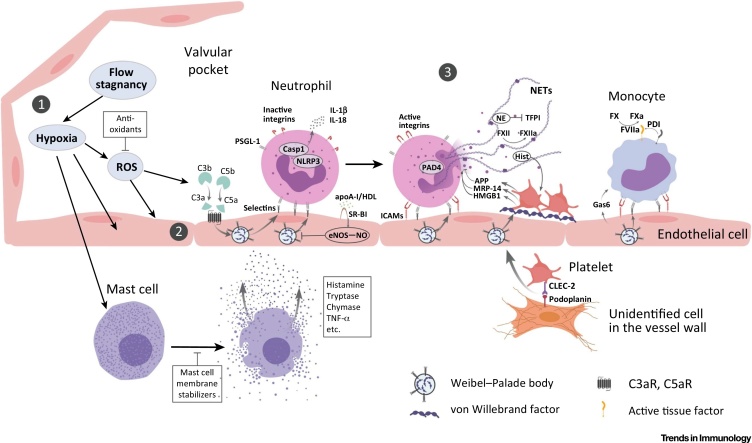
Interplay between Immunological Mechanisms of Deep Vein Thrombosis Initiation. Note that although the inflammasome is depicted in a neutrophil, the exact cell type, in which inflammasomes are formed and IL-1β is synthesized under flow restriction conditions, remains uncertain. Abbreviations: apoA-I, apolipoprotein-AI; APP, amyloid precursor protein; C3, complement component 3; C3aR, C3a receptor; C5, complement component 5; C5aR, C5a receptor; Casp1, caspase-1; CLEC-2, C-type lectin-like receptor-2; eNOS, endothelial NO synthase; FVIIa, activated factor VIIa; FX, factor X, FXa, activated FX; FXII, factor XII; FXIIa, activated FXII; Gas6, growth arrest-specific gene 6; HDL, high-density lipoprotein; Hist, histone; HMGB1, high-mobility group box 1; ICAMs, intercellular adhesion molecules; IL, interleukin; MRP-14, myeloid-related protein-14; NE, neutrophil elastase; NETs, neutrophil extracellular traps; NLRP3, NOD-like receptor family, pyrin domain containing 3; PAD4, peptidyl arginine deiminase 4; PDI, protein disulfide isomerase (an enzyme activating tissue factor); ROS, reactive oxygen species; PSGL-1, P-selectin glycoprotein ligand-1; SR-BI, scavenger receptor BI; TFPI, tissue factor pathway inhibitor; TNF-α, tumor necrosis factor-α.

The initiation of venous thrombus formation involves a complex cascade of events that can be divided into three consecutive though overlapping stages: (i) blood flow stagnancy and hypoxia; (ii) activation of the endothelium; and (iii) blood cell recruitment leading to activation of blood coagulation and thrombus development (Figure 2). We outline these steps and the involvement of immune cells below.

## Stage 1: Blood Flow Stagnancy and Hypoxia Trigger DVT

DVT develops in the valvular pockets of the veins [14]. As blood pressure gradually decreases from the heart ventricles all the way to the veins, the pumping function of the heart may become insufficient to push blood through the veins. Thus, normal function of the auxiliary muscle pump becomes indispensable for proper blood flow especially in the legs given that humans have vertical spinal orientation. When limb muscles do not contract regularly and properly, blood flow velocity in certain veins decreases to complete stasis. This is associated with elevated risk for DVT. Thus, blood flow stagnation in the veins is one of the main factors driving idiopathic DVT (i.e., excluding DVT resulting from a clear reason, such as cancer or trauma) [15]. This is an important stipulation because, for example, DVT caused by cancer (and cancer-associated therapy) is based on elevated blood coagulation induced, in particular, by TF-bearing microparticles [16]. Flow stagnation can result from sessile position of an individual, such as observed after surgery (especially an orthopedic one), in a bed-ridden position or even during long-haul flights. In pediatric patients, immobility for more than 72 h is also considered one of the main factors triggering DVT with each additional day of hospitalization increasing the risk by 3% [17]. Venous stasis increases also with age and it was observed that contrast material stays in the veins of elderly patients for up to 1 h after venography [18]. It should be noted however that blood flow stagnancy either may require additional factors or be unusually prolonged to cause DVT, or both, because, for example, normal night sleep does not result in DVT.

Animal models recapitulating complete [19] or partial 20, 21 blood flow restriction by applying ligature on the inferior vena cava (IVC; stasis and stenosis DVT models, respectively; Table 1) have been developed and used to delineate mechanisms of venous thrombosis initiation and resolution. In addition to the experimental approach, the pivotal role of flow reduction in cell accrual in the valves has recently been demonstrated by a computer simulation approach [22].

Blood inside the vein is the only source of oxygen for the venous wall. Thus, diminished supply with new portions of blood creates local hypoxia in the vein. Hypoxia is considered the major pathogenetic mechanism linking blood flow stagnancy with following processes in the vessel wall leading to thrombosis. Hamer and co-authors directly demonstrated in dogs and patients that blood oxygenation in venous valve pockets quickly falls once the flow becomes static and returns back to original values when pulsatile (i.e., mimicking muscle pump-supported) flow is applied [23]. It has been recently shown that whole-body hypoxia potentiates venous thrombosis 24, 25. Hypoxia results in activation of various cell types in the venous wall, such as mast cells (MCs) and endothelium, leading to expression of adhesion receptors (e.g., intercellular cell adhesion molecule 1, ICAM-1) and release of Weibel–Palade body (WPB; storage granules present in endothelial cells) constituents [26]. This cascade of events is a prerequisite for local recruitment of leukocytes and platelets; an inflammatory phenomenon that was first demonstrated to be induced by flow restriction more than 50 years ago [27]. The recruited cells give start to thrombosis through various routes, some of which are discussed below.

## Stage 2: Endothelial Activation

Endothelial activation and exocytosis of WPBs, central events in DVT initiation, are rapidly (within 1–6 h) induced by stenosis of the IVC 21, 28. In this process, WPBs fuse with the plasma membrane and release their constituents expressing some of them, such as von Willebrand factor (VWF; a coagulation factor present in the WPBs) and P-selectin, on the membrane, thus mediating cell recruitment. This is consistent with increased levels of soluble P-selectin in patients with DVT [29], although soluble P-selectin may originate also from activated platelets. Elevated levels of P-selectin both in the circulation and the venous wall are associated with upregulated DVT in the electrolytic mouse model [30]. Inhibition of P-selectin suppresses DVT and stimulates spontaneous recanalization of the thrombus in an IVC balloon occlusion model in baboons, suggesting its pathophysiological role in venous thrombosis [31].

Hypoxia results in the synthesis of reactive oxygen species (ROS), which activate the endothelium, inducing the expression of adhesion receptors and recruitment and extravasation of leukocytes [32]. ROS may activate endothelial cells also indirectly, for example, via the complement system. Oxidative stress induces complement activation (in particular, by endothelial cells damaged by ROS) and its deposition on the vessel wall leading to progression of inflammation 33, 34. The susceptibility to DVT strongly correlates with complement component C5a (C5a) levels in a mouse model [35]. Mice deficient for C3 or C5 have reduced experimental venous thrombosis, with the lack of C5 not being accompanied by any defects in platelet activation or normal hemostasis [36]. High levels of the C3 are associated with high risk of DVT in humans [37]. Mechanistically, high-molecular-weight multimers of VWF released from WPBs provide a scaffold for complement activation [38]. Complement components bind to VWF strings and become activated through the alternative activation pathway [39]. Thus, in addition to the direct activation of endothelium by hypoxia and ROS, the complement system may represent an additional link between flow restriction and endothelial activation.

In addition to cell recruitment, activated endothelium enhances blood coagulation and exerts suppressed anticoagulant function, thus contributing to thrombus formation [40]. Upregulation of the endothelial surface adhesion receptor ICAM-1 underlies augmented DVT under the endotoxemic conditions [41]. Clinically, endothelial activation has been reported in patients with VTE and thrombosis of superficial veins 42, 43.

It has recently become clear that endothelial activation and cell recruitment, the critical events in DVT initiation likely induced by local hypoxia, require an intermediary: MCs. MCs are a part of the innate immune system that largely reside in tissues and are present in the vicinity of blood vessels [44]. These are large cells containing granules enriched with proinflammatory mediators, such as tumor necrosis factor-α and histamine, and antithrombotic factors, such as tissue plasminogen activator (tPA) and heparin. Thus, MCs might be expected to exert opposite effects on thrombosis: its reduction by releasing blood coagulation inhibitors or stimulation by supporting local inflammatory response. The involvement of MCs in DVT is implicitly supported by their accumulation at the site of venous thrombosis [45], but their net functional contribution has long remained obscure.

Using the stenosis DVT model in mice, we have recently demonstrated that two strains of MC-deficient mice are completely protected against DVT [46]. Adoptive transfer of *in vitro*-differentiated MCs into MC-deficient animals restored thrombosis, suggesting that it is the lack of MCs that prevents DVT. Thus, the net effect of MC degranulation in this model is the exacerbation of venous thrombosis, suggesting that the activity of MC-originated proinflammatory factors outweighs the activity of antithrombotic factors. Absence of MCs completely prevents thrombosis, suggesting that MCs are absolutely required (although may not be sufficient) for its initiation.

In addition to protection from DVT, MC deficiency is accompanied by reduced cell recruitment at the venous wall after stenosis application [46]. Consequently, the factor or factors released from MCs and responsible for DVT may be involved in endothelial activation. Histamine is one of the most likely candidates produced by MCs. Indeed, local application of histamine accelerated DVT in wild-type mice and induced DVT in MC-deficient mice [46]. The mechanisms of the prothrombotic effect of histamine might involve its ability to induce release of VWF and P-selectin from WPBs [47] and enhance expression of E-selectin and ICAM-1 [48], mediating blood cell recruitment to the vascular wall. Histamine also stimulates the expression of TF, the key initiator of blood coagulation, in different cell types 49, 50.

Mechanisms of MC activation by hypoxia remain obscure, but oxidative stress and ROS appear to play a role in this process. ROS can be both the cause and the consequence of MC activation because, on the one hand, inhibition of MC activation downregulates ROS production and, on the other hand, antioxidants prevent MC degranulation [51]. Generation of ROS has a direct impact on DVT in a mouse model. Indeed, H_2_O_2_ has been shown to mediate increased susceptibility to DVT in aged mice, whereas mice overexpressing an antioxidant, glutathione peroxidase 1, are protected against the prothrombotic effect of H_2_O_2_ [52].

Mastocytosis, which is associated with abnormally high numbers of MCs, is accompanied by bleeding symptoms in a small proportion of patients [53]. The reason for this is unclear but it is possible that when the number of MCs exceeds a certain limit, massive release of antithrombotic factors starts to prevail over the effect of proinflammatory/prothrombotic stimuli. However, the importance of MCs in venous thrombosis is corroborated by a clear link between allergic diseases and VTE [54]. Severity of asthma strongly correlates with the risk of not only DVT but also PE [55]. MCs and histamine are implicated in airway and lung inflammation-related thrombosis induced by diesel exhaust particles and prevention of histamine release has an antithrombotic effect [56]. Thus, targeting MCs by inhibiting their activation and degranulation, as well as further identification of and targeting MC granular constituents exacerbating thrombosis, may represent a fundamentally new approach to fight DVT, although the precise benefit and advantages of this approach in patients are still to be verified.

## Stage 3: Blood Cell Recruitment

### Leukocytes

Restriction of venous blood flow induces rapid leukocyte recruitment. After 1 h of IVC stenosis, leukocytes start to roll along and adhere to the venous endothelium, and after 5–6 h, leukocytes carpet the entire endothelial surface. Neutrophils account for more than 80% of adherent leukocytes and monocytes represent the remainder [28]. Leukocyte recruitment is dependent on P-selectin exposure on the luminal side of the venous endothelium since the number of leukocytes recruited to the venous wall in mice lacking P-selectin on the endothelial surface is reduced by several orders of magnitude. Moreover, these mice are protected against DVT, indicating that leukocyte recruitment is crucial for DVT development in response to blood flow restriction. Although activated platelets also expose P-selectin on their surface, the role of platelet-derived P-selectin in leukocyte recruitment is less prominent [28]. Leukocyte recruitment is also affected by various plasma components. For example, higher plasma levels of low-density lipoproteins (LDL) likely enhance leukocyte accumulation since deficiency in proprotein convertase subtilisin/kexin type 9 (PCSK9), an enzymatically inactive protein that binds the LDL receptor favoring its degradation, significantly reduces leukocyte adhesion and thrombus growth in the stenosis DVT model [57]. Given that leukocyte recruitment to the venous wall is indispensable for DVT, below we discuss the contribution of the main leukocyte subsets recruited, namely neutrophils and monocytes, to the pathogenesis of the disease.

### Neutrophils

The involvement of neutrophils in DVT was discovered several decades ago [58]. Recent studies have demonstrated the critical role of neutrophils in the pathophysiology of DVT 28, 59, 60. Depletion of neutrophils inhibits venous thrombus formation, indicating that their role cannot be substituted by other leukocytes [28]. This prothrombotic effect of neutrophils, however, is observed only in the stenosis DVT model, whereas in the stasis model, neutropenia does not affect thrombus size in mice and results in development of even larger thrombi in rats 61, 62.

Upon recruitment to the venous wall, neutrophils undergo activation and release their nuclear material, forming a web-like extracellular structures designated as neutrophil extracellular traps (NETs). These are composed of DNA, histones, secretory granule constituents, and other components implicated in antimicrobial defense [63]. It has been shown that signals originating from the neutrophil P-selectin glycoprotein ligand-1, a counter-receptor for P-selectin, may trigger the process of NET formation (NETosis) [64]. Under pathogen-free conditions of thrombosis, NETosis may also be induced by high-mobility group box 1 (HMGB1) released by and exposed on the surface of platelets recruited to the venous wall 65, 66. Although monocytes may also be able to form extracellular traps [67], experiments on neutropenic mice have shown that neutrophils are the major source of these traps in venous thrombi [28]. NETs are abundantly present in venous thrombi 59, 68, which is in line with increased plasma levels of NETs biomarkers in patients with DVT 69, 70. Prevention of NETosis 71, 72 or destruction of NETs by infusion of deoxyribonuclease (DNase) I 28, 59 protects mice from thrombus formation in the stenosis DVT model, indicating the crucial role of NETs in the onset of DVT. NETs support DVT also in the stasis model in some [66] but not in other studies [73]. The latter study demonstrates that Toll-like receptor (TLR)-9-deficient mice have larger thrombi than control animals, despite elevated levels of NET markers, and that treatment with DNase I or genetic ablation if peptidyl arginine deiminase (PAD)-4 (an enzyme required for NET production) does not reduce thrombus size in the stasis model. More prominent prothrombotic function of neutrophils and NETs in stenosed versus fully closed vessels suggests that residual blood flow is indispensable for the inflammatory mechanism to become operational in venous thrombosis commencement, whereas complete absence of flow likely induces DVT through a more coagulation-dependent mechanism [74].

The mechanisms by which NETs may contribute to venous thrombosis have become an important area of research. It has been shown that various adhesion proteins, including VWF, fibrinogen, and fibronectin, may bind to DNA/histone strings so that NETs become a scaffold for adhering platelets and red blood cells independent of the fibrin network [68]. Upon release into the extracellular space, histones trigger activation of endothelial cells [75], which is consistent with increased plasma levels of VWF in mice infused with purified histones [59]. *In vitro* experiments have also demonstrated that NETs stimulate platelet adhesion and aggregation at a venous shear rate and induce thrombocytopenia *in vivo*, with both effects being abolished by histone inactivation 68, 76.

Another mechanism of the prothrombotic effect of NETs is potentiation of the coagulant cascade and reduction of anticoagulant activity. NETs can bind FXII and provide a scaffold for FXII activation [28]. Activated FXII may amplify fibrin formation without activating FXI, presumably through direct interaction with fibrin [77]. Additionally, neutrophil elastase and other proteases associated with NETs degrade anticoagulants, such as TF pathway inhibitor (TFPI) [78], while histones impair thrombomodulin-dependent protein C activation [79], promoting thrombin generation. A recent study has demonstrated *in vitro* that while NETs components, DNA and histones, potentiate thrombin generation and blood clotting, NETs, as a biological entity, are unable to do so [80]. This implies (if proven *in vivo*) that NETs might need a certain degree of degradation to acquire procoagulant activity. Thus, NETs could represent an important mechanistic link between neutrophil accrual and venous thrombogenesis.

### Monocytes

Monocytes and, to a lesser extent, neutrophils recruited to the venous wall serve as a principal source of TF; the major initiator of the extrinsic coagulation pathway and fibrin deposition. In the stenosis model of DVT, deletion of TF in myeloid leukocytes completely prevents thrombus formation without affecting leukocyte recruitment [28]. In contrast, in the complete stasis model, DVT is driven primarily by the vessel-wall-derived but not leukocyte-derived TF [74]. This difference might be attributed to different pathogenetic mechanisms operating in these similar but distinct models.

The prothrombotic function of leukocytes is negatively regulated by signaling via TLR-9. It has been shown that lack of this pattern-recognition receptor is associated with larger venous thrombi and increased levels of NETosis, necrosis, and apoptosis markers in the stasis, but not stenosis, model of DVT in mice [73]. It has also been shown that lack of TLR-9 leads to reduced monocyte recruitment to venous thrombi [81].

### Platelets

Platelets are recruited to the venous wall shortly after blood flow restriction and play an important role in DVT as platelet depletion substantially reduces thrombosis [28]. A role of platelets in DVT is supported by the observations that an antiplatelet drug aspirin reduces DVT in mice (by preventing thromboxane A2 synthesis) [82] and VTE in patients undergoing orthopedic surgery 83, 84; a condition frequently associated with compromised venous blood flow. Importantly, efficacy of venous thrombosis prophylaxis by aspirin is non-inferior to that of rivaroxaban, an anticoagulant widely used in clinical practice [85], which confirms involvement of platelets in DVT pathogenesis. In contrast to arterial thrombosis, where platelets form large aggregates [78], in DVT, platelets are mainly recruited as single cells and adhere either directly to the activated endothelium or to adherent leukocytes forming small heterotypic aggregates [28].

Platelet recruitment to the venous thrombus is mediated by binding of platelet receptor GPIbα to VWF exposed on the endothelial surface. Indeed, deficiency in either GPIbα extracellular domain [28] or VWF [21] prevents experimental DVT. Recently, it has been shown that platelet recruitment also depends on the platelet membrane molecule CLEC-2, a hemi-immunoreceptor tyrosine-based activation motif-bearing receptor capable of binding podoplanin. Podoplanin is a mucin-type transmembrane protein expressed in the murine IVC wall in tunica media and adventitia (middle and external layers of the venous wall, respectively), and its expression is markedly upregulated in the course of thrombus formation [86]. It has been proposed that hypoxia-induced activation of the endothelial cells, caused by restriction of the blood flow, renders endothelial cell–cell junctions looser, allowing for platelet penetration into subendothelial spaces where the interaction between CLEC-2 and podoplanin may take place [86].

The analysis of signal transduction pathways in platelets following recruitment to the venous wall has shown a role for mechanistic target of rapamycin complex 1 (mTORC1), a rapamycin-sensitive protein complex consisting of mTOR, Raptor, and mLST8 (mammalian lethal with SEC13 protein 8) [87]. Deficiency of mTORC1 considerably reduces DVT in the murine flow restriction model. Platelet recruitment to the developing venous thrombus is also associated with enhanced generation of ROS, promoting thrombus growth [52]. The contribution of both mTORC1 and ROS to the pathogenesis of DVT increases with age 52, 87, which is consistent with higher incidence of VTE in elderly patients [88]. Platelet recruitment and DVT in the conditions of hypobaric hypoxia, such as encountered at high altitude, depend in a mouse model also on assembly of NOD-like receptor family, pyrin domain containing 3 (NLRP3) inflammasome [25], a molecular platform triggering autoactivation of caspase-1, which cleaves the proinflammatory cytokines, interleukin (IL)-1β, and IL-18, into their active forms (reviewed in [89]). Deficiency in NLRP3 is associated with reduced thrombus size in complete stasis-induced DVT in mice [25]. This finding is in accordance with the study demonstrating increased serum IL-18 levels in experimental DVT in rats [90] as well as with the clinical observation demonstrating increased levels of IL-1β and IL-18 in patients with DVT [25].

Besides procoagulant activity (reviewed in [91]), recruited platelets provide important proinflammatory stimuli being a source of various damage-associated molecular patterns (DAMPs) 92, 93. Following recruitment to the venous wall, platelets expose HMGB1 [92], a nucleosomal protein that serves as a DAMP when released into the extracellular space. Deficiency in platelet-derived HMGB1 markedly decreases thrombus size and thrombosis incidence in the DVT model [65]. Operating through the receptor for advanced glycation end-products (RAGE) and other pattern recognition receptors, HMGB1 promotes NETosis of the recruited neutrophils and facilitates recruitment of monocytes [65]; an important source of TF triggering the extrinsic coagulation pathway. Additionally, HMGB1 promotes recruitment and activation of new platelets at early stages of venous thrombus formation. Enhanced NETosis and platelet accrual result in further HMGB1 accumulation in the developing thrombus forming a positive feedback propagating DVT [65]. Myeloid-related protein (MRP)-14, a member of the S100 family of calcium-modulated proteins, is another DAMP abundantly expressed in platelets and neutrophils [94]. MRP-14 deficiency is associated with reduced DVT, which is partially rescued by adoptive transfer of wild-type platelets or neutrophils. Acting in a Mac-1-dependent manner, MRP-14 fosters NETosis, thereby promoting venous thrombus propagation. Collectively, the data characterize platelets as important regulators of sterile inflammation and stress the role of platelet–neutrophil crosstalk in venous thrombogenesis.

Involvement of platelets in the pathogenesis of DVT is limited by several mechanisms. Platelet recruitment to the venous endothelium is downregulated by the interaction of apoA-I, the major apolipoprotein in high-density lipoprotein (HDL), with endothelial receptor scavenger receptor-BI (SR-BI). This interaction diminishes endothelial activation and WPB release in an endothelial NO synthase (eNOS)-dependent manner [95]. SR-BI-mediated signaling protects from venous thrombosis in mice, which is consistent with increased risk of DVT in patients with low plasma HDL levels [96], although some reports contradict this [97]. The ability of platelets to promote leukocyte recruitment to the venous thrombus is negatively regulated by amyloid precursor protein (APP) abundantly expressed in platelets. In the stasis DVT model, substitution of wild-type platelets by APP-deficient ones increased platelet–leukocyte interaction. Genetic deficiency in APP is associated with enhanced NETosis, greater incorporation of NETs into venous thrombi, and enhanced DVT [98]. Thus, APP or its functional analogs may represent a new approach to DVT prevention targeting simultaneously local inflammation and NETs production.

## Concluding Remarks

Mechanisms of DVT initiation represent a cascade of events virtually identical to the local inflammatory response recently designated as immunothrombosis [99]. This opens a window of opportunities for identification of new antithrombotic targets because (i) the immune system is not directly implicated in normal hemostasis and targeting it is unlikely to result in excessive bleeding; and (ii) multiple anti-inflammatory drugs are already on the market and, consequently, available for testing their efficacy to prevent DVT.

Although the precise mechanisms of how the immune-system-related cells and molecules are implicated in DVT may differ, some of them converge on local inflammation in the venous wall. Thus, focusing on this common denominator, reduction of endothelial activation, release of WPBs and local cell recruitment could be a promising strategy ameliorating venous thrombosis. For example, NO is one of the most potent inhibitors of WPB liberation [100], and we can therefore speculate about potential usefulness of NO donors (which are already in clinical use) for DVT prevention. HDL activates eNOS through binding SR-BI [101] and a component of HDL, apoA-I, was shown to efficiently reduce DVT in experimental conditions operating via the same route [95]. A mutated form of apoA-I with higher lipid-binding propensity, called apoA-I Milano, downregulates arterial thrombosis caused by ferric chloride [102]. Of note, synthetic apoA-I analogs have been developed and proven to recapitulate various effects of the natural protein [103]. Hence, the apoA-I/HDL axis might be considered potentially useful to fight DVT. Histones, a part of NETs, also stimulate WPB release [104] and their prothrombotic activity can therefore rely, at least in part, on this effect. Based on experimental evidence, endothelial activation can be limited also by targeting MC degranulation, especially given that drugs with such mechanism of action are already in clinical use for other purposes. Determination of targetable mechanisms of MC activation in the unique environment of the veins, identification of the MC-derived factors promoting DVT, and verification of the relevance and efficacy of this approach in patients represent a challenge for future research. Thus, the following directions currently seem to be most promising in the translational aspect to prevent DVT by manipulating the immune system: (i) amelioration of local vessel response to hypoxia; (ii) inhibition of endothelial activation and WPB release (e.g., NO, targeting MC etc.); (iii) inhibition of immune cell recruitment (e.g., adhesion receptor antagonists); and (iv) targeting NETs.

In conclusion, DVT develops as a form of immunothrombosis with a particularly important role of local inflammation at the stage of thrombosis initiation. Targeting inflammatory pathways is less likely to cause bleeding complications than inhibition of blood coagulation mechanisms. Thus, it may be considered as an alternative and safer approach for the prevention of DVT in at-risk populations and further research in this area may provide important new therapeutic options (See Outstanding Questions).

Outstanding Questions Is local hypoxia a leading factor exacerbating DVT? Are there (if any) other causes?

What are the mechanisms of hypoxia-driven local inflammation?

What mechanisms mediate mast cell activation and degranulation under flow stagnancy conditions? What mediators released by mast cells trigger DVT?

What therapeutic interventions targeting the immune system and local inflammation will be most efficient against DVT?

Based on combination of targeting the immune response with conventional methods of tackling DVT, it is tempting to develop a personalized approach of DVT prevention in different predisposing conditions (e.g., infection/sepsis, allergy, cancer, or major surgery).
